# Report of envenomation in humans by handling a dyeing poison frog *Dendrobates tinctorius* (SCHNEIDER, 1799) (Anura: Dendrobatidae) in the Amazon, Brazil

**DOI:** 10.1590/0037-8682-0461-2020

**Published:** 2020-12-21

**Authors:** Bruno Alessandro Augusto Peña Corrêa, Vitor Matheus Alcântara de Sena, Rubens Hisanari Matsushita, Nathalie Kaladinsky Citeli

**Affiliations:** 1 Universidade de Brasília, Laboratório de Anatomia Comparada, Brasília, DF, Brasil.; 2 Universidade de Brasília, Laboratório de Planejamento para a Conservação da Biodiversidade, Brasília, DF, Brasil.; 3 Freelance Researcher, Brasil.; 4 Universidade de Brasília, Laboratório de Fauna e Unidades de Conservação, Brasília, DF, Brasil.

**Keywords:** Amphibians, Injuries, Hypesthesia

## Abstract

We report a case of envenomation by *Dendrobates tinctorius* in the northwest of the Amazon Forest. The patients were two men, who presented with numbness in the right arm and slight numbness in the lower lip, respectively. *Dendrobates tinctorius* secretions contain pumiliotoxin, one of several toxins found in the dendrobatidis skin, which interferes with muscle contraction and causes locomotor difficulties. Although Dendrobatidae is a family of anurans known for their venom, few studies describe the symptoms of envenomation in humans.

## INTRODUCTION

Amphibians have a range of defense mechanisms against predators, among which is the presence of toxins in their skin^1^. Such poisons can be produced by the animals themselves or obtained from their food (e.g., anurans of the family Dendrobatidae), and can be stored along its whole body or in specific regions such as the paratoid glands in some species (e.g., toads of the family Bufonidae and salamanders)^1^ ^,^ ^2^. The Dendrobatidae family comprises 202 species of aposematic anurans^3^. Aposematic refers to an animal which uses colors or shapes to warn potential predators that they have defense mechanisms, such as venoms^1^. Their skin toxins can be composed of several distinct classes of alkaloid compounds (batrachotoxins, histrionicotoxins, pumiliotoxin C, and the pumiliotoxin A family, gephyrotoxins, and a group of indolicizidines)^4^ ^,^ ^5^.

Alkaloids are basic chemical compounds, made from amines, which are found mainly in plants, and can be sequestered by animals such as ants, bettles, termites, and other invertebrates^6^ ^,^ ^7^. The Dendrobatidae family’s specialized diet in ants allows for the acquisition of alkaloids for defense mechanisms and the evolution of aposematic forms^6^. The dyeing poison frog *Dendrobates tinctorius* (Cuvier, 1797), is a medium-sized frog that can be found in open areas of the Amazon Forest. It is a diurnal species with a wide occurrence in Central America, French Guiana, Suriname, and in the north of Brazil^8^. The main alkaloid carried by this species is pumiliotoxin (PTX), which is highly toxic in high concentrations^4^. PTX interferes with muscle contraction by affecting calcium channels, causing locomotor difficulties, clonic convulsions, paralysis, or even death, depending on the affected organism^4^. Here, we report and describe the symptoms of two human envenomations by *Dendrobates tinctorius*.

## CASE REPORT

**FIGURE 1:**
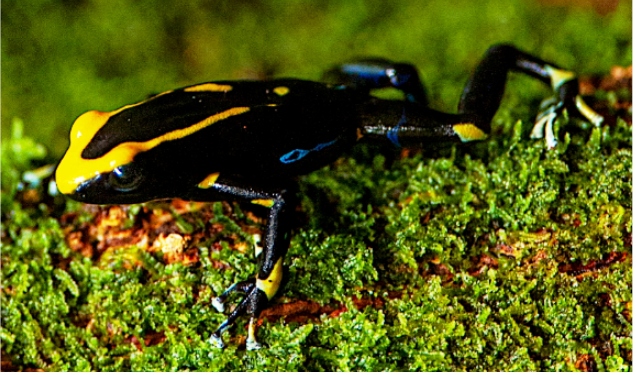
*Dendrobates tinctorius* specimen handled by the photographers.

The incident occurred on 4th August 2013 at 11:40 a.m., near the city of Monte Dourado, Pará, Brazil. Two photographers, 47 and 30 years old, were walking on a trail when one of them spotted a *D. tinctorius* (Figure 1) next to a tree trunk. Even though they identified the frog as being from the Dendrobatidae family, they touched it so they would be able to photograph it. The first photographer quickly captured the frog and held it for about five seconds with his own hands, before releasing it and washing his hands in a nearby stream. The other photographer then kept the frog from moving by placing both his hands on top. Neither of the photographers suffered hand injuries.

Both men photographed the tree-frog for about five minutes, without touching it again. However, 20 minutes after the first contact with the anuran, the photographer who initially handled it began to feel numbness in his right arm, mainly at the height of the forearm.

The other photographer, who after taking photos had touched his mouth without first washing his hands, felt a slight numbness in his lower lip. The pair reported that they did not seek medical attention because the numbness was mild and after about 40 min they no longer felt symptoms. Species identification was carried out by Dr. Carlos Eduardo Costa de Campos, professor at the Federal University of Amapá (Herpetology Laboratory), and the tree frog was not collected.

## DISCUSSION

Accidents involving amphibians in Brazil are not listed by the Brazilian Ministry of Health in its list of human intoxications^9^. Uncommon, medically relevant accidents involving humans and anurans occur with field workers or due to the increasing use of frog’s toxins by inexperienced practitioners in rituals, such as “kambô”^10^ ^,^ ^11^.

In our report, the photographer’s symptoms could have been aggravated if contact with the animal had lasted longer or if there had been a wound at the points of contact. 

The *D. tinctorius* toxin can lead to cardio-respiratory problems, mainly through the neurotoxic action of the poison, which affects the sodium and potassium channels, impairing muscle contraction and, consequently, the heart and breathing muscles^4^. 

Although anurans in this family are considered a health risk, there are few studies on the substances found in the skin of *D*. *tinctorius* and even fewer studies on their physiology^7^. For example, approximately 37% of the alkaloids found in Dendrobatidae are unclassified, with over 250 alkaloids of unknown structural class awaiting chemical characterization^2^ ^,^ ^7^. 

Regardless, the handling of this species requires special attention, and the risk of potential envenomation should be taken into account. In case of accidents, we strongly recommend reporting to health authorities so that possible side effects and negative outcomes can be clearly documented.
